# Multidisciplinary approach and treatment of acral and mucosal melanoma

**DOI:** 10.3389/fonc.2024.1340408

**Published:** 2024-02-26

**Authors:** Ana Fortuna, Teresa Amaral

**Affiliations:** ^1^ Oncology Department, Centro Hospitalar Universitário do Algarve, Faro, Portugal; ^2^ Center for Dermatooncology, Department of Dermatology, Eberhard Karls University of Tübingen, Tübingen, Germany; ^3^ Cluster of Excellence Image-Guided and Functionally Instructed Tumor Therapies (iFIT) (EXC 2180), Tübingen, Germany

**Keywords:** acral melanoma, mucosal melanoma, KIT mutations, GNAQ mutation, GNA11 mutation, immunotherapy, targeted therapy, local therapy

## Abstract

Acral and mucosal melanoma are uncommon variants of melanoma. Acral melanoma has an age-adjusted incidence of approximately 1.8 cases per million individuals per year, accounting for about 2% to 3% of all melanoma cases. On the other hand, mucosal melanoma, with an incidence of 2.2 cases per million per year, makes up around 1.3% of all melanoma cases. These melanomas, in addition to being biologically and clinically distinct from cutaneous melanoma, share certain clinical and pathologic characteristics. These include a more aggressive nature and a less favorable prognosis. Furthermore, they exhibit a different mutational pattern, with KIT mutations being more prevalent in acral and mucosal melanomas. This divergence in mutational patterns may partially account for the relatively poorer prognosis, particularly to immune checkpoint inhibitors. This review explores various aspects of acral and mucosal melanoma, including their clinical presentation, pathologic features, mutational profiles, current therapeutic approaches, outcomes associated with systemic therapy, and potential strategies to address resistance to existing treatments.

## 1 Introduction

Acral and mucosal melanoma are rare types of melanoma. Acral melanoma (AM) accounts for approximately 2% to 3% of all melanomas, with an age-adjusted incidence of 1.8 cases per million people per year (1). Mucosal melanoma (MM) accounts for 1.3% of all melanomas, with an incidence of 2.2 cases per million per year (2). Individuals with higher phototypes in Latin America and Africa predominantly develop AM, whereas MM is more predominant in Asian individuals (3).

AM affects the palms, soles, and nails and tends to be more advanced at time of diagnosis. The histopathologic subtype of most, but not all, AM is acral lentiginous. Acral lentiginous melanoma is composed of cells that occur as single units along the dermal-epidermal junction and as confluent foci. This form of melanoma is most commonly found in acral sites. MM arises from mucous membranes such as the mouth, nasopharynx, larynx, conjunctiva, vagina, or anus (4).

Diagnosis is usually made late in the course of the disease, particularly in the case of AM, which may be mistaken for traumatic warts. MM is also often diagnosed late because their most common location makes them difficult for patients to recognize it.

Due to their localization, AM and MM are less likely to be associated with ultraviolet (UV)-exposure and therefore have a lower mutation burden. Typical mutations present in cutaneous melanoma (CM) are BRAF (v-raf murine sarcoma viral oncogene homolog B1) V600E (found in approximately 50% of the cases), but BRAF mutations are found in only 10-35% of AM and 0-21% of MM (5, 6). In contrast, KIT mutation is found in approximately 3-36% of AM and 7-25% of MM, and 5-10% of CM overall carry KIT mutations (5).

The decision-making process for treating AM and MM should involve a multidisciplinary team. Primary treatment for these melanomas typically involves surgery in the early stages. However, due to the unique localization and, in some cases, challenging surgical requirements, it’s crucial to consider treatment in specialized centers. In more advanced stages of AM and MM, treatment primarily includes systemic therapy, namely immunotherapy, targeted therapy, and chemotherapy. Additionally, in specific cases, radiotherapy may be incorporated into the treatment plan.

This review provides a comprehensive overview of the epidemiology, diagnosis, and current therapeutic strategies for AM and MM. It underscores the importance of a multidisciplinary evaluation in managing these melanomas effectively. It places particular emphasis on clinical trial whose results are reported by tumor subtype. Furthermore, it highlights the potential benefits of including patients with AM and MM in future clinical trials, rather than excluding them deliberately.

## 2 Epidemiology

AM accounts for a greater proportion of melanomas in Black, Asian, and Hispanic populations compared to only 4-6% in Caucasians, due to the low prevalence of UV-induced melanoma in these ethnic groups (7). It is typically diagnosed at an average age of 65 years. The incidence of AM increases with age in all populations, with a significant increase in incidence per person-year after the age of 80. In contrast to CM, where men are more commonly affected, AM affects both sexes equally (8). The 5-year overall survival (OS) rate for AM is 80.3% compared to 91.3% for all CM (p <.001) (1).

MM accounts for less than 1% of all malignant melanomas. It is most commonly found in the head and neck region, followed by the anus and rectum, female reproductive tract, and, less commonly, the urinary tract mucosa (9). They are more frequently observed in Asia, Africa, and some countries in Latin America (3). The average age at diagnosis is around 70 years, compared to 65 years in CM, and women are twice as likely to be affected (9). A study of 161 patients with MM found that patients younger than 60 years old had better survival rates, but advanced stages of the disease had a significant negative impact on survival (10). MM is one of the most aggressive subtypes of melanoma, with a 5-year OS rate of 10-20%. Due to its rarity, no risk factors have been identified to date (9).

Although rare, acral and mucosal melanoma are the dominant subtypes in China and other Asian countries, accounting for 50% and 20-30% of cases, respectively (11).

## 3 Diagnosis and staging

AM is difficult to diagnose as it can be easily confused with other dermatologic conditions and the sites of origin are often less visible. Therefore, it is usually diagnosed at a later stage. It is not yet clear whether this late diagnosis leads to a worse outcome or whether AM is inherently more aggressive (12). MM can also be difficult to diagnose accurately because it resembles benign lesions and occurs in less commonly examined anatomical sites (13).

Melanoma diagnosis can be delayed due to various factors, including socioeconomic and cultural factors (7, 14). Patient factors, such as lack of knowledge and routine skin examination, are the most significant contributors to delays (15). Additionally, low socioeconomic status has been linked to advanced stage at presentation and poor prognosis in melanoma patients (7, 16, 17). There are significant differences among racial and ethnic groups in psychological and cultural factors such as language, beliefs, and knowledge about cancer (18). These factors may contribute to the disproportionate cancer burden among patients belonging to minorities and are important to consider when addressing cancer disparities (14, 18). Although the role of these factors in minority patients has not been clearly defined, studies have shown that low socioeconomic status independently predicts poor outcomes in the general melanoma population (7, 17).

Although socioeconomic and cultural factors are not modifiable risk factors in the short term, improving health literacy can influence health attitudes and behaviors. Therefore, this should be considered in the development of public health strategies (19).

Medical history, full-body skin examination and dermoscopy by dermatologists are widely considered to be the best methods for early diagnosis of AM and MM. These methods significantly improve the diagnostic accuracy compared to other examination methods (4, 12).

## 4 Mutational landscape

The genetic profile of acral and mucosal melanoma is significantly different from the genetic profile of UV-induced melanoma. Therefore, oncogenic signaling pathways are not reliant on UV damage and differ from those observed in CM. This is reflected in the presence of different significantly mutated genes, lower mutational burdens, more structural variants, and fewer mutations induced by UV radiation (refer to **Table 1**) (5, 6, 20).

**Table 1:** Mutational landscape in acral and mucosal melanoma.

Pathway	Gene	Frequency in cutaneous melanoma (%)	Frequency in acral melanoma (%)	Frequency in mucosal melanoma (%)
**MAPK**	BRAF mutations	42-50 [5,20–22]	10-35 [5,20,22]	0-21 [5,20–24]
	RAS mutations	28-30 [5,20,22]	8-22 [5,20–22]	5-25 [5,20–22,24]
	NF1 mutations	10-23 [5,20,22]	11-26 [5,20,22]	0-34 [20–22,24]
	Triple wild-type	5-10 [5,20,22]	32-58 [5,20,22]	37-75 [5,20,22,24]
	c-KIT aberrations	5-10 [5,20–22]	3-36 [5,20,22]	7-39 [5,20–22,24]
	GNAQ mutations	1.5-2.1 [5,22]	0-17 [5,22]	1-12 [5,22,24]
	GNA11 mutations	Rare [5,22]	Rare [5,22]	1 [5,22,24]
	MAP2K1 and MAP2K2 mutations	2-4 [5,20,22]	5-8 [5,20,22]	0-11 [5,20,22,23]
	SPRED1 mutation or loss	5 [20]	1 [20]	8-26 [20,21]
**Cell cycle**	CDKN2A mutation	13-40 [5,20,22]	0-3 [5,20,22]	Rare [5,20,22–24]
	CDKN2A loss	45 [5,22]	35 [5,22]	10-38 [5,22,23]
	RB1 mutation	4-15 [5,20,22]	9-17 [5,22]	0-21 [5,20,22,23]
	TP53 mutation	15-27 [5,20,22]	2-54 [5,20,22]	7-15 [5,20,22,23]
	CCND1 mutation	1-13 [5,20,22]	5-54 [5,22,30][5,22]	25 [5,22]
	CCND1 amplifications	–	45.4 [30]	27.7 [34]
	CDK4 mutation or gain	5-6 [5,22]	9 [5,22]	5-25 [5,22]
	CDK4 amplifications	–	–	47 [34]
	P16^INK4a^ deletion	–	–	57.7 [34]
**PI3K/AKT**	PTEN mutation or loss	8.5-40 [5,20,22]	7-28 [5,20,22]	1-25 [5,20,22–24]
	mTOR mutations	6.7 [35]	11 [35]	–
**Telomerase**	TERT promoter mutations or gain	52-85 [5,20,22]	5-45 [5,20,22]	5-42 [5,20,22–24]
**Copy numbers variation**	CDK4 - CNV	–	19.3 [32]	–
	CCND1 - CNV		19.3 [32]	–
	MDM2 - CNV		14.5 [32]	–
	FGF19 - CNV		12 [32]	–

CNV, copy number variation.

### 4.1 Driver mutations related to the MAPK (mitogen-activated protein kinase) pathway

The identification of driver mutations that lead to carcinogenesis, in contrast to passenger mutations, is particularly challenging in CM due to the high mutation burden caused by the mutagenic effects of UV radiation (20). Acral and mucosal melanomas exhibit significantly lower mutational burdens compared to CM (5, 6, 20).

The intracellular signaling pathway known as the MAPK pathway is crucial and frequently activated in melanoma, promoting tumorigenesis (21). Approximately 42-50% of the CM are BRAF mutants, mainly due to mutations in the V600 codon (5, 20–22). BRAF mutations were identified in 10-35% of AM and 0-21% of MM (5, 20–24). However, BRAF V600 mutations are present in only 6% of MM cases (21).

About 30% of CM cases are RAS (rat sarcoma) mutants, with mutations in either NRAS (neuroblastoma-RAS) (primarily at codon Q61), KRAS (Kirsten-RAS), or HRAS (Harvey-RAS) (5, 20, 22). However, RAS mutations are less common in AM and MM, occurring in only 8-22% and 5-25% of cases, respectively (5, 21, 22, 24). The prevalence of NF1 (Neurofibromatosis Type 1) mutations is approximately 10-23% in CM, 11-26% in AM, and 0-34% in MM (5, 20–22, 24). A proportion of 5-10% of CM are triple wild-type, while in AM and MM, the proportion is higher, with 32-58% and 37-75%, respectively (5, 20, 22, 24).

KIT mutations are activators of both the MAPK and PI3K (Phosphoinositide 3-kinase)/AKT (protein kinase B) pathways and are more frequent in AM and MM than in CM, where the frequency is 5-10% (5, 20–22). The frequency of c-KIT aberrations is 3-36% in AM and 7-39% in MM (5, 20–22, 24). It is estimated that around 10-15% of patients with AM and MM have KIT mutations. Therefore, clinical features should be considered when deciding whether to test for BRAF and/or KIT mutations (5, 25). Similar to KIT, fes-related (FER) receptor tyrosine kinase amplification is often associated with NF1 inactivation, but not with BRAF and NRAS alterations. This suggests a potential role for FER amplification in driving the MAPK pathway (6).

In CM, mutations in GNAQ (guanine nucleotide-binding protein G(q) subunit alpha) are found in 1.5-2.1%, while mutations in GNA11 (guanine nucleotide-binding protein G(q) subunit alpha 11) are infrequent (5, 22). In the case of AM, mutations in GNAQ are present in only about 0-17% of cases, and mutations in GNA11 are also rare (5, 22). In MM, approximately 1-12% of patients have mutations in GNAQ, and around 1% have mutations in GNA11 (5, 22, 24). However, mutations in GNAQ or GNA11 have been found in around 10% of Chinese patients with MM, and are associated with a worse prognosis. These mutations are believed to be among the factors that contribute to the poorer survival of Chinese patients with MM compared to Caucasian patients (26).

Mutations in MAP2K (mitogen-activated protein kinase kinase) 1 and MAP2K2 are found in approximately 2-4% of cases of CM, 5-8% of cases of AM, and 0-11% of cases of MM (5, 20, 22, 23). Additional studies have demonstrated the association of the rs2228230:T SNP in Platelet-Derived Growth Factor Receptor Alpha (PDGFRA) with decreased PDGFRA expression, downstream signaling activity, and improved survival in AM patients (27). Furthermore, AM has a higher probability of having mutations in genes that are associated with the MAPK pathway including C-KIT and PDGFRA, when compared to CM (28).

SPRED1 (sprouty-related, EVH1 domain-containing protein 1) loss was detected in 26% of MM cases. Additionally, a significant association between SPRED1 loss and KIT alterations was discovered in 30% of cases. *In vitro* and *in vivo* models showed that SPRED1 loss, in association with KIT mutations, led to increased MAPK pathway activity and conferred resistance to the KIT tyrosine kinase inhibitor dasatinib. These findings establish SPRED1 as a possible tumor suppressor gene that collaborates with activating KIT mutations to maintain MAPK signaling and may therefore confer resistance to KIT inhibition. However, the impact of SPRED1 loss in MM has yet to be fully defined (21).

Approximately 94% of CM carry mutations that activate the MAPK pathway, such as BRAF, NRAS, and NF1, as opposed to only 28% of MM cases (21). Moreover, besides BRAF fusion, hotspot mutations in MAP2K1 and KRAS oncogenes have also been identified in MM, implying further dependence on activating the MAPK pathway (25). Additionally, MM has been reported to have mutations in CTNNB1 (catenin beta-1). These mutations impact the activation of the Wnt/β-catenin pathway and may be a contributing factor to the inadequate response to immunotherapy among patients with advanced MM (29).

### 4.2 Driver mutations related to the cell cycle

In CM, CDKN2A (cyclin-dependent kinase inhibitor 2A) mutation and loss are present in about 13-40% and 45%, respectively (5, 20, 22). The CDKN2A mutation is infrequent in AM, ranging from 0-3%, and is rarely observed in MM (5, 20, 22–24). However, CDKN2A loss is present in 35% of AM cases and in 10-38% of MM cases (5, 22, 23). Tumors with a higher number of CDKN2A deletions are significantly associated with a worse prognosis (5).

RB1 (retinoblastoma 1) mutations occur in 4-15% of cutaneous, 9-17% of acral, and 0-21% of mucosal melanomas (5, 20, 22, 23). TP53 mutations are present in 15-27% of CM, but are more common in AM at 2-54% and in MM at 7-15% (5, 20, 22, 23).

Mutations in CCND1 (cyclin D1) are found in 5-13% of CM (5, 22). However, in a study of 44 Chinese patients with AM, multiple fluorescence *in situ* hybridization analyses revealed that 45.4% of them had CCND1 amplification, while mutations in CCND1 were found in only 5-24% (5, 22, 30). CCND1 is mutated in 25% of MM patients (5, 22). In both AM and MM, patients with CCND1 amplification had better outcomes (6).

Compared to CM, AM has a higher number of copy number variations (CNV) in Caucasians, in contrast to a Chinese cohort (5, 22, 31). Indeed, CNV in AM were common in CCND1 (19.3%), CDK4 (cyclin-dependent kinase 4) (19.3%), MDM2 (mouse double minute 2 homolog) (14.5%), and FGF19 (fibroblast growth factor 19) (12%). Moreover, CDK4 amplifications were independently linked to shorter OS (32). Similarly, frequent alterations in the CDK4 signaling pathway, which facilitates the G1 to S-cell cycle transition and tumor progression, were found in a study of 514 cases of AM. Importantly, genetic mutations in CDK4, CCND1, or P16^INK4a^ (cyclin-dependent kinase inhibitor 2A) were found in 82.7% of the samples (33). Another study found that mutations in genes linked to the cell cycle were prevalent, with amplifications in CDK4 and CCND1 present in 47.0% and 27.7% of samples, respectively, while P16^INK4a^ was deleted in 57.7% of MM cases. Therefore, alterations in CDK4 signaling components may predict response to CDK4 inhibitors in MM (34).

### 4.3 Driver mutations related to the PI3K/AKT pathway

PTEN (phosphatase and tensin homolog) mutation or loss is present in up to 8.5-40% of CM, 7-28% of AM, and 1-25% of MM (5, 20, 22–24). In both AM and MM, patients with TP53 mutations had worse outcomes. In addition, a PTEN alteration was associated with a worse survival rate in MM (6).

Mutations in mTOR (mammalian target of rapamycin) can also be present in melanoma. Non-synonymous mTOR mutations were found in 10.4% of 412 melanomas. Of note, compared to chronic sun-damaged melanoma (6.7%), mTOR mutations were more common in AM (11.0%). PI3K-AKT-mTOR pathway inhibitors present a promising therapeutic option for patients with mTOR-mutated melanoma; non-synonymous mTOR mutations have been strongly linked to reduced survival in melanoma patients (35).

### 4.4 Driver mutations related to the telomerase pathway

Telomerase reverse transcriptase (TERT) mutations play an important role in the development of melanoma. TERT promoter mutations are present in 52-85% of CM cases, whereas they only occur in approximately 5-45% of AM cases and 5-42% of MM cases (5, 20, 22–24). Furthermore, TERT promoter mutations are less common in acral and mucosal melanomas, representing only about 11% of all cases (22).

### 4.5 Chromosomal aberrations

The AM and MM subtypes showed a higher frequency of certain features, including all structural variants (deletions, duplications, tandem duplications, and foldback inversions), as well as an increased occurrence of breakpoint clusters. These clusters indicate a higher incidence of complex structural rearrangements, such as breakage-fusion-bridge (BFB) and chromothripsis (22, 36).

In the genomic landscape of 87 AM, structural rearrangement and copy number signatures indicate the prevalence of whole genome duplication, aneuploidy, and complex rearrangements. The recurrent complex rearrangements are associated with the amplification of TERT, CDK4, MDM2, CCND1, PAK1 (p21-activated kinase 1), and GAB2 (Grb2-associated binder 2). These alterations could be potential targets for systemic therapies (36).

### 4.6 Others

A study of whole-genome sequences from cutaneous, acral, and mucosal subtypes of melanoma in 183 melanoma samples found that BRAF, NRAS, and NF1 were significantly mutated genes in AM, while SF3B1 (splicing factor 3B subunit 1) was identified in MM (22, 36). Furthermore, these subtypes presented various ‘triple wild-type’ mechanisms, including KIT mutations and focal amplifications of KIT, CCND1, MDM2, and KRAS. Neither acral nor mucosal melanomas had mutations in TP53, PTEN, DDX3X, RASA2, PPP6C, RAC1, or RB1. Moreover, TP53, PTEN, and RB1 pathway defects were not notable drivers of these melanoma subtypes (22). These subtypes also have a lower tumor burden than CM (5, 22, 37).

An understanding of the genomic characteristics of acral and mucosal melanoma may aid in the understanding of their pathogenesis and the development of potential preventive and therapeutic strategies. Acral and mucosal melanomas develop on areas of the body that are partially or completely shielded from environmental UV radiation, in contrast to CM, which frequently occur on sun-exposed skin. Both subtypes of melanoma share similar genomic characteristics, including a low somatic mutation burden and an increased number of copy number and structural alterations. There is only a partial overlap between the genetic alterations found in acral and mucosal melanoma and those found in CM. For instance, BRAF mutations are less frequent in acral and mucosal melanomas. However, the prevalence of KIT mutations, CCND1, CDK4, and MDM2 amplifications, and SPRED1 deletions is higher. The landscape of somatic and germline driver events remains incompletely characterized due to their rarity (5, 6, 20–22, 25).

## 5 Treatment of localized and locoregional disease

The management of localized AM and MM closely parallels that of CM. The gold standard treatment for patients with clinically negative regional lymph nodes in these cases is wide surgical excision. It’s essential that this procedure is carried out in a specialized surgical center to ensure optimal care.

In the case of AM, surgical resection frequently necessitates plastic surgical reconstruction to preserve aesthetics and functionality after extensive excision. Achieving a complete resection with negative margins can be challenging in many cases of MM. This challenge is often attributed to the location of the tumor, particularly in areas such as the paranasal sinuses, or the fact that it may present as multifocal or with poorly defined margins due to a lentiginous growth pattern, resulting in a high local recurrence rate of 50-90% (38).

For patients with primary AM <0.8 mm with ulceration or > 0.8 - 1.0 mm (with or without ulceration), sentinel lymph node biopsy is usually recommended (39), similar to other CM. As for MM, the prognostic value of sentinel lymph nodes has not been established. Complete regional lymph node dissection following a positive sentinel node is also a matter of debate in patients with MM (40).

Adjuvant radiation therapy may be considered for select patients with AM or MM to improve locoregional control. However, its effect on OS has not been established (41, 42).

## 6 Systemic treatment

Limited evidence exists from large international cohort studies on the effectiveness of various systemic treatments for acral and mucosal melanoma due to their rarity. In comparison to other melanoma subtypes, targeted therapy and immunotherapy have limited efficacy in AM and MM due to the lack of dominant MAPK activating mutations that can be targeted and the lower response to immunotherapy (38). Therefore, standardized and effective systemic therapies represent an unmet clinical need.

### 6.1 Immunotherapy

#### 6.1.1 Advanced stage

In advanced cases of CM, there is strong support for the use of therapy with immune checkpoint inhibitors, particularly anti-programmed cell death protein 1 (anti-PD-1) therapy. This can be given either alone or in combination with the anti-cytotoxic T lymphocyte antigen*-*4 (CTLA-4) antibody ipilimumab. CheckMate 066, 067, and KEYNOTE-006 are among several clinical trials that have shown survival benefit of treatment with immune checkpoint inhibitors (ICI). However, there is no information available regarding the benefit for each subtype of melanoma (see **Table 2**) (43, 44, 50).

**Table 2:** Clinical trials for Advanced Treatment in Melanoma.

Reference	Phase	Stage	Arms	Patients	Outcomes	
					Primary endpoint(s)	In AM and MM
1^st^ line						
**CheckMate 066** (43)	III	Unresectable stage III or IV	Group A: nivolumab 3 mg/kg Q2W	Total: 518 AM: NA MM: NA	Median OS: not reached	NA
			Group B: dacarbazine 1000 mg/m^2^ Q3W		Median OS: 10.8 months (95% CI, 9.3 to 12.1)	
**CheckMate 067** (44)	III	Unresectable stage III or IV	Group A: nivolumab 1 mg/kg + ipilimumab 3 mg/kg Q3W for 4 doses, followed by nivolumab 3 mg/kg Q2W	Total: 945 AM: NA MM: NA	Median PFS: 11.5 months (95% CI, 8.9 to 16.7) OS: Data not matured	NA
			Group B: nivolumab 3 mg/kg Q2W		Median PFS: 6.9 months (95% CI, 4.3 to 9.5)	
			Group C: ipilimumab 3 mg/kg Q3W for 4 doses followed by placebo Q2W		Median PFS: 2.9 months (95% CI, 2.8 to 3.4)	
**Nomura M, et al.** (45)	II	Unresectable or metastatic MM	Nivolumab 2 mg/kg every 3 weeks	Total: 20 AM: 0 MM: 20	ORR: 23.5%	
**RELATIVITY 047** (46)	II/III	Advanced	Nivolumab 480mg and relatlimab 160 mg, Q4W	Total: 714 AM: 41 MM: 23	Median PFS 10.1 months	AM and MM had better outcomes with relatlimab-nivolumab
			Nivolumab 480mg, Q4W		Median PFS: 4.6 months	
**Hodi FS, et al.** (47)	II	Metastatic mucosal, acral, or chronically sun-damaged melanoma with *KIT* aberrations	Imatinib	Total: 24 AM: 6 MM: 17	ORR: 29% (two-stage 95% CI, 13% to 51%)	ORR (AM): 0% ORR (MM): 41%
**Robert C, et al.** (48)	III	IIIC, IV	Dabrafenib and trametinib	Total: 704 AM: NA MM: NA	Median OS: not reached 1 year OS: 72% (95% CI, 67 to 77)	NA
			Vemurafenib		Median OS: 17.2 months 1 year OS: 65% (95% CI, 59 to 70)	
**Guo J, et al.** (49)	II	*KIT*-mutated advanced melanoma	Nilotinib	Total: 42 AM: 20 MM: 20	ORR: 26.2% (95% CI, 13.9%–42.0%)	NA
≥ 1^st^ line						
**KEYNOTE-006** (50)	III	Unresectable stage III or IV	Group A: pembrolizumab 10 mg/kg Q2W	Total: 834 AM: NA MM: AN	Median PFS: 5.5 months (95% CI, 3.4 to 6.9) 1 year OS: 74.1%	NA
			Group B: pembrolizumab 10 mg/kg Q3W		Median PFS: 4.1 months (95% CI, 2.9 to 6.9) 1 year OS: 68.4%	
			Group C: ipilimumab 3 mg/kg Q3W × 4 doses		Median PFS: 2.8 months (95% CI, 2.8 to 2.9) 1 year OS: 58.2%	
**POLARIS-01** (51)	II	Advanced Chinese who had failed in systemic treatments	Toripalimab: 3 mg/kg Q2W	Total: 128 AM: 50 MM: 22	ORR: 17.3% (95% CI 11.2–25.0) per RECIST v1.1 and 18.1% (95% CI 11.8–25.9) per irRECIST	ORR (AM): 14% ORR (MM): 0%
**KEYNOTE-151** (52)	Ib	Advanced (previously treatment with one line of therapy)	Pembrolizumab 2 mg/kg Q3W	Total: 103 AM: 39 MM: 15	ORR: 6.7% (95% CI, 10.0%-25.3%)	ORR (AM): 15.8% ORR (MM): 13.3%
**CheckMate 172** (53)	II	Advanced (progression on or after ipilimumab)	Nivolumab 3 mg/kg Q2W	Total: 1008 AM: 55 MM: 63	Incidence of grade ≥3 adverse events: 18.2%	Median OS (AM): 25.8 months (95% CI, 15.1-30.6) Median OS (MM): 11.5 months (95% CI, 6.4-15.0)
**D’Angelo SP, et al.** (54)	Pooled analysis	Unresectable stage III or IV, MM	Nivolumab monotherapy	Total: 157 AM: 0 MM: 157	Median PFS: 3.0 months (95% CI, 2.2 to 5.4 months) ORR: 23.3% (95% CI, 14.8% to 33.6%)	
			Nivolumab combined with ipilimumab		Median PFS: 5.9 months (95% CI, 2.8 months to not reached) ORR: 37.1% (95% CI, 21.5% to 55.1%)	
			Ipilimumab monotherapy		Median PFS: 2.7 months (95% CI, 2.6 to 2.8 months) ORR: 8.3% (95% CI, 1.8% to 22.5%)	
**Hamid O, et al.** (55)	*Post-hoc* analysis	Unresectable stage III or IV	Pembrolizumab 2 mg/kg every 3 weeks (Q3W) or 10 mg/kg Q2W or Q3W	Total: 1567 AM: NA MM: 84	Non-MM: median PFS: 4.2 months (3.6-5.5) Median OS: 23.5 months (21.1-26.8) ORR: 33% (95% CI 30-35%)	MM: Median PFS: 2.8 months (95% CI 2.7-2.8) Median OS: 11.3 months (95% CI 7.7-16.6) ORR: 19% (95% CI 11-29%)
**KEYNOTE-041** (56)	Ib	Unresectable stage III) or metastatic stage IV with 0–2 prior lines of therapy for advanced melanoma	Pembrolizumab 2mg/kg Q3W	Total: 42 AM: 12 MM: 8	ORR: 24.3%, 11.8–41.2	ORR (AM): 25.0% ORR (MM): 25.0%, 3.2–65.1
**Guo J, et al.** (57)	II	Metastatic melanoma with c-Kit aberrations	Imatinib	Total: 43 AM: 21 MM: 11	6-month PFS: 36.6% median PFS: 3.5 months (range, 1.3 to 5.7 months)	NA
**Carvajal R, et al.** (58)	II	Melanoma with KIT mutations or amplification	Nilotinib	Total:19 AM: 4 MM: 12	Median time to progression: 3.3 months (90% CI, 2.1 - 3.9 months) OS: 9.1 months (90% CI, 4.3 - 14.2 months)	NA
**Lee SJ, et al.** (59)	II	Metastatic melanoma with KIT mutations or amplification	Nilotinib	Total: 42 AM: 21 MM: 12	ORR: 16.7% (95% CI: 5.4%−28.0%) DCR: 57.1% (95% CI: 42.1%−72.1%)	NA
**Mao L, et al.** (60)	IIa	Unresectable or metastatic BRAF V600-mutant AM or CM	Dabrafenib plus trametinib	Total: 60 AM: 12 MM: NA	ORR: 71.7%	ORR (AM): 83.3%
Retrospective analysis						
**Nakamura Y, et al.** (61)	Retrospective study	Unresectable stage III or stage IV AM	Anti-PD-1 antibody (nivolumab or pembrolizumab)	Total: 193 AM: 193 MM: 0	ORR (AM): 16.6% PFS (AM): 3.5 months OS (AM): 18.2 months	
**Bhave P, et al.** (62)	Retrospective study	Unresectable stage III/IV AM	Anti-PD-1	Total: 325 AM: 325 MM: 0	ORR: 26%	
			Anti-PD-1/ipilimumab combination		ORR: 43%	
			Ipilimumab		ORR: 15%	
**Nakamura Y, et al.** (63)	Retrospective study	Advanced AM	Anti-PD-1 monotherapy and anti-PD-1 plus anti-CTLA-4 combination	Total: 254 AM: 254 MM: 0	AM - palm and sole melanoma: ORR: 19% (monotherapy) *vs*. 31% (combination); P = 0.44) PFS: 5.9 *vs*. 3.2 months; P = 0.74 OS: 23.1 *vs*. not reached; P = 0.55 AM - nail apparatus melanoma: ORR: 10% *vs*. 61%; P < 0.001 PFS: 3.8 *vs*. 6.4 months; P = 0.10	
**Nakamura Y, et al.** (64)	Retrospective study	Advanced MM	Anti-PD-1 monotherapy or combination with anti-PD-1 and anti-CTLA4	Total: 329 AM: 0 MM: 329	ORR: 26% (monotherapy) versus 29% (combination therapy); *P* = 0.26 Median PFS: 5.9 (monotherapy) versus 6.8 months (combination therapy); *P* = 0.55 Median OS: 20.4 (monotherapy) versus 20.1 months (combination therapy); *P* = 0.55	
**Dimitriou F, et al.** (65)	Retrospective study	Metastatic or unresectable MM	Anti- PD-1 monotherapy or combination anti-PD-1 and ± ipilimumab	Total: 545 AM: 0 MM: 545	ORR: 30% (95% CI, 26-34%) Median PFS: 4 months (95% CI, 4-6 months) Median OS: 19 months (95% CI, 18-24 months)	
**Bai X, et al.** (66)	Retrospective study	Metastatic or unresectable BRAF-mutant AM and MM	BRAF inhibition	Total: 40 AM: 28 MM: 12	median PFS (AM): 3.6 months (95%CI 3.0-6.4) months median OS (AM): 6.2 months (95%CI 6.1-12.1) ORR (AM): 38.1% median PFS (MM): 4.4 months (95%CI 0.8-12.7) median OS (MM): 8.2 months (95%CI 6.6-19.9) ORR (MM): 20.0%	
**Wei X, et al.** (67)	Retrospective study	Advanced melanoma with *c-Kit* mutations or amplifications	Imatinib	Total: 78 AM: 42 MM: 16	median OS: 13.1 months (95% CI: 9.6–16.7 months) median PFS: 4.2 months (95% CI: 1.9–6.4 months) ORR: 21.8%	NA
**Fujosawa Y, et al.** (68)	Retrospective study	Advanced	BRAF and MEK inhibitor	Total: 112 AM: 11 MM: 3	Response rate in CM: 76.5% (67.1%- 83.5%) median PFS: 13.0 months (95% CI: 6.0-21.8) median OS 35.0 months (95% CI: 16.0 to not reached)	Response rate in AM and MM: 64.3% (42.9% to 78.6%)

AM, acral melanoma; CI, confidence interval; DCR, disease control rate; MM, mucosal melanoma; NA, not available; OS, overall survival; ORR, overall response rate; PFS, progression-free survival.

A multicenter phase II trial of toripalimab, a humanized anti-PD-1 antibody, was conducted in 128 Chinese patients diagnosed with melanoma (POLARIS-01), including 50 patients with AM and 22 with MM. The ORR for patients with AM and MM treated with toripalimab was 14.0% and 0%, respectively. Median OS for AM and MM were reported to be 16.9 months and 10.3 months, respectively. Median progression free survival (PFS) for AM and MM was 3.2 months and 1.9 months, respectively. Additionally, low response to toripalimab treatment was associated with NRAS mutations and CCDN1 amplifications, which are common in Chinese melanoma patients. These findings likely reflect immunotherapy resistance in the Asian group, which had higher KIT mutation rates in addition to lower PD-L1 expression and tumor mutational burden (51).

The phase Ib KEYNOTE-151 study evaluated pembrolizumab as a second-line treatment for advanced melanoma. The study enrolled patients with AM (37.9%) and MM (14.6%). The ORR for patients with acral and mucosal subtypes were similar at 15.8% (95% CI, 6.0-31.3%) and 13.3% (95% CI, 1.7-40.5%), respectively (52). The phase II CheckMate 172 study evaluated the efficacy of nivolumab in 55 patients with unresectable AM who had experienced disease progression or recurrence after prior treatment with anti-CTLA-4 monoclonal antibodies. The median OS was 25.8 months (95% CI, 15.1-30.6), which was similar to that of patients with CM (25.3 months; 95% CI, 20.9-28.9) (53).

A retrospective evaluation of 193 patients with unresectable stage III or IV AM receiving any line of anti-PD-1 therapy showed an ORR of 16.6% and median OS of 18.1 months. The results suggest that the efficacy of anti-PD-1 treatment in patients with advanced melanoma is limited (61). A retrospective analysis of 325 patients with unresectable stage III/IV AM treated with anti-PD-1 and/or ipilimumab showed an ORR to first-line ICI of 26% for anti-PD-1, 45% for anti-PD-1 plus ipilimumab and 12% for ipilimumab. The longest PFS was 5.4 months (95% CI, 3.4-11.7) for the combination of anti-PD-1 and ipilimumab, compared with 4.1 months (95% CI, 3.7-5.9) for anti-PD-1 and 3.5 months (95% CI, 2.9-4.1) for ipilimumab. Regarding OS benefit, the median OS was 1.9 years for patients receiving anti-PD-1 (95% CI 1.4-2.6%) monotherapy, 1.3 years for patients receiving anti-PD-1 plus ipilimumab (95% CI 1.2-2.7), and 1.9 years for patients treated with ipilimumab (95% CI 1.3-2.6) (62).

Another retrospective study was conducted in 254 Japanese patients diagnosed with AM and treated with first-line anti-PD-1 inhibitors or anti-PD-1 in combination with anti-CTLA-4. The efficacy of the combination was not superior to that of anti-PD-1 alone for the treatment of advanced palm and sole melanoma, as the ORR was 19% *vs*. 31% (p = 0.44), PFS 5.9 *vs*. 3.2 months (p = 0.74), and OS 23.1 *vs*. not reached (p = 0.55), respectively. However, the treatment of advanced nail apparatus melanoma in the combination arm showed a significantly higher objective response rate (ORR) (61% *vs*. 10%, p<0.001) and longer PFS (6.4 months) compared to anti-PD-1 alone (3.8 months; p = 0.10). Therefore, it appears that treatment with this combination may be more effective than anti-PD-1 therapy alone in this location. Anti-PD-1 and anti-CTLA-4 combination was an independent predictor of favorable PFS in nail apparatus melanoma patients in Cox multivariate analysis (63).

A pooled analysis of six clinical trials was conducted to evaluate the effectiveness and safety of nivolumab monotherapy or its combination with ipilimumab for treating unresectable stage III or IV MM. Data from patients enrolled in the trials CA209-003, CA209-038, CheckMate 066, CheckMate 037, CheckMate 067 and CheckMate 069 were included. This analysis involved 86 MM patients who received nivolumab monotherapy, 35 patients who were treated with nivolumab combined with ipilimumab, and 36 patients who received ipilimumab monotherapy. The median PFS was 3.0 months (95% CI, 2.2-5.4 months) for those receiving nivolumab monotherapy, 5.9 months (95% CI, 2.2-not reached) for those receiving combination therapy, and 2.7 months (95% CI, 2.6-2.8 months) for those receiving ipilimumab monotherapy. ORR were lower in all MM groups compared to the CM group (54). In MM, the combination of nivolumab and ipilimumab showed greater efficacy than monotherapy.

Other pooled analysis was conducted to assess pembrolizumab efficacy in advanced MM patients, including patients treated in the KEYNOTE-001 (69), 002 (70) and 006 (50) studies. With a median follow-up of 27.6 months, the ORR was 19% (95% CI, 11-29%). The median PFS was 2.8 months (95% CI, 2.7 to 2.8), and OS was 11.3 months (95% CI, 7.7-16.6) indicating less favorable outcomes in comparison to non-MM (55). In another phase II trial involving 20 patients with advanced MM who received nivolumab treatment, similar unfavorable outcomes were reported – ORR of 23.5%, median PFS of 1.4 months (95% CI, 1.2-2.8), and median OS of 12.0 months (95% CI, 3.5-not reached) (45).

However, Asian patients appear to have worse clinical outcomes than Caucasians. The Japanese Mucosal Melanoma (JMAC) study retrospectively assessed 329 patients with advanced MM who were treated with anti-PD1 monotherapy (nivolumab or pembrolizumab) or a combination of anti-PD1 and anti-CTLA4. The study found that both monotherapy and combination therapy had a similar ORR of 26% and 29%, respectively. Additionally, both treatments had similar PFS with median PFS of 5.9 months and 6.8 months, respectively and similar OS with median OS of 20.4 months and 20.1 months, respectively. The study also found no evidence of prolonged PFS and OS with the use of anti-PD1 and anti-CTLA4 (64). The Phase Ib Keynote 041 study evaluated the safety of pembrolizumab in Japanese patients with advanced melanoma and demonstrated an ORR of 25% in MM (56).

Finally, a retrospective study was conducted in 545 patients with metastatic or unresectable MM who were treated with anti-PD-1 monotherapy (nivolumab or pembrolizumab) or a combination of anti-PD-1 and CTLA4, ipilimumab. The ORR was similar in the two groups (29% in the PD-1 monotherapy group and 31% in the anti-PD-1/ipilimumab combination group). Median PFS and OS were also comparable between anti-PD-1 monotherapy (5 and 19 months, respectively) and the anti-PD-1/ipilimumab combination (4 and 21 months, respectively) (65).

#### 6.1.2 Adjuvant setting

Adjuvant therapy should be considered for patients with stage IIB or higher who have undergone complete surgical resection. However, there is a lack of robust evidence for the benefit of adjuvant therapy in patients with AM and MM as these tumor subtypes are underrepresented in the registration studies (see **Table 3**). In The KEYNOTE-716 study of pembrolizumab in the adjuvant setting for high-risk stage IIB and IIC node-negative disease, a statistically significant reduction in the risk of recurrence was observed (71). The CheckMate 76K trial, which evaluated the benefit of adjuvant nivolumab therapy in the same setting, enrolled 43 patients with AM, 28 in the nivolumab group and 15 in the placebo group. Patients with MM were excluded and no subgroup analysis considering histologic subtype was performed (72). Checkmate 238 compared nivolumab with ipilimumab following complete resection of stage IIIB, IIIC or IV melanoma. A total of 33 patients diagnosed with AM and 29 patients with MM were enrolled, but in the subgroup analysis, the magnitude of the benefit of the different treatments for each melanoma subtype was inconclusive due to the small number of patients enrolled in each subgroup (73).

**Table 3:** Clinical trials for the adjuvant treatment of melanoma.

Reference	Phase	Stage	Arms	Patients	Outcomes	
					Primary endpoints	In AM and MM
**KEYNOTE-716** (71)	III	IIB-C	Pembrolizumab 200 mg or every 3 weeks for 17 cycles or until disease recurrence or unacceptable toxicity	Total: 1182 AM: NA MM: NA	RFS: not reached in either group	NA
			Placebo			
**CheckMate 76K** (72)	III	IIB-C	Nivolumab 480 mg every 4 weeks for 12 months	Total: 790 AM: 43 MM: NA	12-month RFS: 89.0% (95% CI: 85.6–91.6)	NA
			Placebo		12-month RFS: 79.4% (95% CI: 73.5–84.1)	
**CheckMate 238** (73)	III	IIIB-C, IV	Group A: nivolumab 3 mg/kg Q2W	Total: 906 AM: 33 MM: 29	12-month RFS: 70.5% (95% CI, 66.1 to 74.5)	No clear benefit of what type of adjuvant treatment
			Group B: ipilimumab 10 mg/kg Q3W 4 doses then every 12 weeks beginning at week 24 for up to 1 year		12-month RFS: 60.8% (95% CI, 56.0 to 65.2)	
**Jacques SK, et al.** (74)	Retrospective study	Resected stage III or IV AM or MM	Adjuvant treatment with anti-PD-1 versus matched historical data	Total: 157 AM: 116 MM: 41	Median RFS (AM): 17.35 months (adjuvant therapy) *vs* 17.4 months (database matched) Median DMFS: (AM): 31.95 months (adjuvant therapy) *vs* 25.9 months (database matched) Median RFS (MM): 18.8 months (adjuvant therapy) *vs* 11.65 months (database matched) Median DMFS: (MM): 23.65 months (adjuvant therapy) *vs* 14.5 months (database matched)	
**Muto Y, et al.** (75)	Retrospective study	Postoperative melanoma patients who received adjuvant therapy	Anti-PD-1 monotherapy (nivolumab or pembrolizumab) in the adjuvant setting	Total: 78 AM: 31 MM: 11	RFS: 60.3% (95% CI, 49.2–70.4%)	RFS (AM): 25.8% (95% CI 14.5–43.5%)
**EORTC 1325/KEYNOTE-054 trial** (76)	III	IIIA-B-C	Group A: pembrolizumab 200 mg Q3W	Total: 1019 AM: NA MM: NA	5 years RFS: 55.4% (95% CI, 50.8 to 59.8)	NA
			Group B: placebo		5 years RFS: 38.3% (95% CI, 33.9 to 42.7)	
**COMBI-AD** (77)	III	IIIA-B-C, BRAF V600 mutant	Group A: dabrafenib 150 mg twice a day + trametinib 2 mg once daily	Total: 870 AM: NA MM: NA	3 years RFS: 58%	NA
			Group B: double placebo		3 years RFS: 39%	

AM, acral melanoma; CI, confidence interval; DMFS, distant metastases-free survival; MM, mucosal melanoma; NA, not available; RFS, recurrence-free survival.

In a retrospective study of 157 patients, 116 (74%) with AM and 41 (26%) with MM, the median time to relapse was 17.7 months for AM patients and 12.9 months for MM patients. The authors concluded that resected AM and MM are associated with a poor prognosis regardless of the use of adjuvant anti-PD-1 therapy. In terms of recurrence-free survival and distant metastasis-free survival, no apparent benefit of adjuvant anti-PD1 was observed compared to historical controls (74).

A retrospective analysis of 78 Japanese patients with advanced melanoma, included 31 cases (40%) of AM and 11 cases (14%) of MM, on anti-PD-1 monotherapy in the adjuvant setting in an Asian population. The relapse-free survival rate was 60.3% in the overall group and 25.8% in the AM group, while there was no significant difference between MM and CM. In this study, AM was found to be an independent prognostic factor (75). However, due to the small sample size and short follow-up period, it remains unclear whether adjuvant ICI truly improves survival outcomes.

#### 6.1.3 Neoadjuvant setting

Despite receiving the best available standard of care, patients with locoregionally advanced but surgically operable melanoma still face a high risk of recurrence and death. New targeted and immunotherapy drugs and combinations in the neoadjuvant setting are being rapidly introduced and showing significant clinical promise.

The neoadjuvant approach has been tested in patients with stage III and oligometastatic resectable stage IV melanoma (see **Table 4**). The Southwest Oncology Group (SWOG) S1801 trial analyzed the efficacy of pembrolizumab as a peri-operative regimen, with three cycles of pembrolizumab before surgery, followed by pembrolizumab adjuvant, in total 1 year of therapy, compared to 1 year of adjuvant therapy alone. In this trial, there were a total of 9 patients with AM, 4 in the neoadjuvant-adjuvant arm and 5 in the adjuvant control arm, and a total of 4 patients with MM, all in the neoadjuvant-adjuvant arm. After 14.7 months of follow-up, 7 out of 9 AM patients and all MM patients were alive, with the two deaths occurring in the adjuvant-only group (81). The combination of neoadjuvant nivolumab-relatlimab was investigated in patients with resectable clinical stage III or oligometastatic stage IV melanoma. The study included patients with both AM and MM, although the precise number of patients enrolled with these subtypes was not reported (80). Neoadjuvant therapy with nivolumab and ipilimumab was also evaluated in high-risk macroscopic node-positive disease in the OpACIN-neo and PRADO clinical trials. However, these phase II trials did not specify the melanoma subtypes of the population included (78, 84, 85).

**Table 4:** Clinical trials for neoadjuvant treatment for resectable melanoma.

Reference	Phase	Stage	Arms	Patients	Outcomes	
					Primary endpoint(s)	In AM and MM
**OpACIN-neo** (78)	II	III with macroscopic lymph node metastases	Group A: 2 doses of ipilimumab 3 mg/kg plus nivolumab 1 mg/kg Q3W followed by surgery	Total: 86 AM: NA MM: NA	ROR: 63% [95% CI 44-80] Pathological response: 80% [61 to 92]	NA
			Group B: 2 doses of ipilimumab 1 mg/kg plus nivolumab 3 mg/kg Q3W followed by surgery		ROR: 57% [37–75] Pathological response: 77% [58 to 90]	
			Group C: 2 doses of ipilimumab 3 mg/kg Q3W plus 2 doses of nivolumab 3 mg/kg Q2W followed by surgery		ROR: 35% [17-56] Pathological response: 65% [44 to 83]	
**Cui C, et al.** (79)	II	Localized or regional lymph node metastasis MM	Toripalimab 3 mg/kg Q2W plus axitinib 5 mg BID for 8 weeks, followed by surgery, and then toripalimab for totally 52 weeks	Total: 21 AM: 0 MM: 21	Pathologic response rate: 28.6%	
**Amaria R, et al.** (80)	II	IIIB-C, IV	2 doses of nivolumab 480 mg and relatlimab 160 mg Q4W, followed by surgery, and then 10 doses of adjuvant combination therapy	Total: 30 AM: NA MM: NA	pCR: 57%	NA
**SWOG S1801** (81)	II	IIIB-C, IV	Neoadjuvant–adjuvant group: 3 doses of pembrolizumab (200 mg Q3W), surgery, and 15 doses of adjuvant pembrolizumab	Total: 313 AM: 9 MM: 4	2 years EFS: 72% (95% CI, 64 to 80)	NA
			Adjuvant-only group: surgery followed by pembrolizumab (200 mg Q3W for a total of 18 doses)		2 years EFS: 49% (95% CI, 41 to 59)	
**NeoCombi** (82)	II	IIIB-C	Dabrafenib plus trametinib for 12 weeks, followed by surgery, then 40 weeks of adjuvant therapy	Total: 35 AM: NA MM: NA	RECIST response: 86% pCR: 49%	NA
**Amaria R, et al.** (83)	II	IIIB-C, IV	Dabrafenib and trametinib for 8 weeks, followed by surgery, then up to 44 weeks of adjuvant dabrafenib plus trametinib	Total: 21 AM: 1 MM: NA	EFS: 19·7 months [16·2 to not estimable]	NA
			Standard of care group: Upfront surgery and consideration for adjuvant therapy		EFS: ·9 months [95% CI 1·7 to not estimable]	

AM, acral melanoma; CI, confidence interval; EFS, event-free survival; MM, mucosal melanoma; NA, not available; pCR, pathologic complete response; RECIST, Response Evaluation Criteria in Solid Tumors; ROR, Radiological objective response

In a phase II study, the neoadjuvant combination of axitinib and toripalimab, an anti-PD-1 antibody, was evaluated in 21 patients with resectable MM. Results showed a pathological response rate of 28.6%, median recurrence-free survival (RFS) of 55.7 weeks, and good tolerability (79).

ICI has been successful in treating CM, but response rates are lower in patients with acral and mucosal melanoma. There is a lack of robust data on neoadjuvant immunotherapy for AM and MM due to the limited number of patients enrolled in trials and even their exclusion from these trials. Thus, it is not possible to make conclusive statements about the efficacy of neoadjuvant immunotherapy for these types of melanoma.

### 6.2 Targeted therapies

#### 6.2.1 Advanced stage

##### 6.2.1.1 BRAF/MEK inhibitors

In patients with advanced BRAF-mutated melanoma, the combination of BRAF and MEK inhibitors has demonstrated significant improvement in both OS and PFS (see **Table 2**) (86–92). However, no information regarding the inclusion of specific melanoma subtypes is available (48). The clinical effectiveness of BRAF and MEK inhibitors in AM and MM is constrained by their unique mutational landscapes, and the efficacy of these inhibitors in this particular group is limited by the relatively low frequency of BRAF mutations.

Patients with BRAF V600-mutated AM and MM showed response rates comparable to those with CM with response rates of 64.3% and 76.5%, respectively, according to a study conducted in Japan (68). Another phase II trial conducted in China with 12 patients with AM treated with dabrafenib plus trametinib showed an ORR of 83.3% and a 3-year OS rate of 35.7% (60). In a retrospective analysis including data from 40 patients with AM (n=28) and MM (n=12), the ORR to BRAF inhibitors (including vemurafenib, sorafenib, and BGB-283 – still in phase I clinical trial) was 38.1% and 20%, respectively (66). Thus, in patients with AM and MM harboring BRAF mutations, combination therapy with BRAF and MEK inhibitors should be discussed.Patients with advanced melanoma harboring NRAS mutations at diagnosis have significantly shorter OS. Due to the technical difficulties of targeting RAS, researchers have shifted their focus to targeting various downstream RAS effectors. In RAS-mutated cancer, MEK inhibitors have been used with limited success (93). A consistent decrease in the expression of phosphorylated extracellular signal-regulated kinase (pERK), a predicted biomarker of MAPK inhibition, was observed in a biomarker study of MEK inhibition with binimetinib in patients with NRAS-mutated melanoma (94). Some phase II studies have shown some effect in CM. The phase III NEMO trial included patients with advanced unresectable stage IIIc or IV melanoma (patients with MM were excluded) who were previously untreated or had progressed on ICI and were treated with either binimetinib or dacarbazine. Median PFS was longer in the binimetinib arm, and patients who had received prior immunotherapy also had longer median PFS with binimetinib compared to those who received dacarbazine (5.5 months [2.8-7.6] *vs*. 1.6 months [1.5-2.8]) (95). Information on the inclusion and outcomes of AM was not provided.

In a phase I/II trial, tunlametinib or HL-085, a selective MEK inhibitor, was well-tolerated with manageable side-effects and showed promising anti-cancer activity. The median PFS was 17.4 weeks and the best ORR was 33.3% in patients with NRAS-mutated advanced melanoma, including acral (51.4%) and mucosal (27.2%) subtypes (96). A phase I study was conducted to evaluate the safety and efficacy of HL-085 in patients with advanced NRAS-mutated melanoma, including 54.8% AM and 31.0% MM patients. The ORR was 14.3% (95% CI: 5.4-28.5%), and the median PFS was 3.0 months (95% CI: 2.1-3.7) (97).

Monotherapy with MEK inhibitors has limited clinical efficacy and a short duration of response due to the emergence of drug resistance resulting from on-target reactivation of the MAPK and PI3K-AKT pathways. New combined therapies are needed to overcome this resistance. In a phase Ib escalation/expansion study, naporafenib was combined with trametinib to treat patients with previously treated NRAS-mutated melanoma. The study showed promising clinical response with an ORR of 30%, median PFS of 5.03 months, and disease control rate (DCR) of 73.3% (98). Further developments in this area are pending.

##### 6.2.1.2 KIT inhibitors

Compared to CM, KIT mutations and/or amplifications have shown to be most commonly associated with AM and MM (99). At least two KIT inhibitors have been investigated as treatment option for patients with advanced melanoma – imatinib and nilotinib.

A phase II study of 24 patients with metastatic melanoma (17 with MM and 6 with AM) harboring activating KIT mutations or amplifications found that imatinib had an ORR of 29% (95% CI, 13-51%). While it is important to note that only MM exhibited a response, there were no significant differences in response observed between the different melanoma subtypes. Treatment with imatinib may be useful if tumors harbor KIT mutations rather than just KIT amplifications. Conversely, NRAS mutations and KIT copy number gain can be associated with resistance to imatinib (47).

A single-arm phase II study was conducted in China to evaluate the effectiveness of imatinib in 43 patients with metastatic melanoma and c-Kit aberrations, comprising 21 patients with AM and 11 with MM (57). The median PFS was 3.5 months and the 6-months PFS rate was 36.6%. The ORR was 23.3%, and the 1-year OS rate was 51.0%. The study also showed that increasing the dose to 800 mg/d after disease progression did not induce further disease control (57).

A retrospective analysis conducted in 78 patients with metastatic melanoma treated with imatinib, including 42 with AM and 16 with MM harboring c-Kit mutation or amplification reported an ORR of 21.8%, a PFS of 4.2 months and a median OS of 13.1 months (67).

Several phase II studies have also evaluated nilotinib, another KIT inhibitor. A phase II clinical trial evaluated the efficacy of nilotinib in 19 participants diagnosed with melanoma harboring KIT mutations or amplifications, 11 refractory or intolerant to a prior KIT inhibitor. The study included 4 patients with AM and 12 patients with MM. The study showed a median time to progression of 3.3 months (90% CI, 2.1 to 3.9 months) and OS of 9.1 months (90% CI, 4.3 to 14.2 months). The results suggest that nilotinib may provide disease control in melanoma patients with KIT alterations whose disease has progressed after treatment with imatinib (58).

Another phase II trial included 42 patients with KIT-mutated advanced melanoma, with 20 having AM and 20 MM patients. Based on the results, nilotinib may be a treatment option for patients with certain KIT mutations. Nilotinib demonstrated greater efficacy compared to imatinib, particularly in patients with an exon 11 mutation, encompassing L576P (49). Another phase II study recruited 42 patients with metastatic melanoma and KIT mutations or amplifications, including 21 with AM and 12 with MM. The study demonstrated lasting responses, with an ORR of 16.7% and a DCR of 57.1% (59).

In a retrospective study of 38 patients with KIT-mutated melanoma treated with imatinib, 6 had AM, and 25 had MM. The ORR was 25% in AM and 38% in MM. Meanwhile, the median PFS was 4.5 and 2.7 months and the median OS was 18.0 and 21.8 months, respectively. MM was associated with a longer PFS, whilst exon 17 mutations were linked to a shorter PFS (100). According to a phase II trial involving 52 patients with unresectable stage III or IV AM or MM, sunitinib exhibited an overall DCR of 44%, along with a high rate of treatment-related toxicity. At 2 months, 52% of patients were alive and progression-free (95% CI, 38%-66%) (101).

##### 6.2.1.3 CDK4/6 inhibitors

The high incidence of CDK4/CCND1 mutations and amplifications in AM suggests that CDK4/6 inhibitors may be a treatment option for patients with this melanoma subtype. In a Phase II study in 15 patients with advanced AM who had failed or were intolerant to at least one prior therapy or who refused standard therapy and had CDK4 and/or CCND1 gain or CDKN2A loss, oral palbociclib treatment resulted in a median PFS of 2.2 months and a median OS of 9.5 months. Patients with amplification of MCM7 or high levels of its protein were more likely to benefit from palbociclib treatment (102).

##### 6.2.1.4 Combination therapies

In a single-arm Phase II study involving 15 advanced recurrent melanoma patients, including those with AM or MM, researchers evaluated the efficacy of apatinib. The median PFS was 4.0 months, the median OS was 12.0 months, and the DCR was 86.7%. In patients diagnosed with malignant melanoma, apatinib exhibited anti-tumor efficacy with manageable toxicity as a second-line or later treatment option (103).

A single-arm phase II study of apatinib in combination with camrelizumab was conducted on 30 patients with first-line metastatic AM. The study reported a median PFS of 7.39 months (CI: 3.65-9.92) and a median OS of 13.4 months (CI: 1.9-25.0). The ORR and DCR was 24.1% and 82.8%, respectively (16). A real-world study found a response rate (RR) of 28%, DCR of 38%, median PFS of 3 months, and median survival of 11 months with lenvatinib and pembrolizumab in advanced metastatic melanoma, including 6 MM and 4 AM (104).

The use of PI3K/Akt/mTOR, CDK, or MDM2/p53 inhibitors for the treatment of AM and MM is currently being investigated (105–107). Driver mutations in NRAS, NF1, CTNNB1, and amplifications in CDK4 have been described in MM, however therapies targeting effectively targeting these alterations are missing (108).

Another potential therapeutic target is the ERBB2 amplification, which is observed in about 3% of AM and MM. In one case report, a complete response to trastuzumab emtansine was found in a patient with ERBB2-amplified AM, who was resistant to ICI (109). Additionally, TRK inhibitors may provide benefits in treating MM, such as those with NTRK3 fusions (110).

A phase IB trial investigated the safety and efficacy of axitinib and toripalimab, a humanized anti-PD-1 antibody, in 29 Asian patients with untreated metastatic MM. The investigation demonstrated good tolerability and promising clinical activity. The combination of axitinib and toripalimab presents a potentially promising treatment option for MM based on current research findings (111).

#### 6.2.2 Adjuvant setting

Despite the development of targeted therapy in melanoma, no adjuvant therapies have been clinically evaluated for acral or mucosal melanoma.

#### 6.2.3 Neoadjuvant setting

##### 6.2.3.1 BRAF/MEK inhibitors

Neoadjuvant targeted therapy with BRAF plus MEK inhibitors, namely dabrafenib and trametinib was also investigated in patients harboring BRAFV600 mutations. One study, NeoCombi, involved 35 patients with resectable stage IIIB-C melanoma, but did not specify the melanoma subtypes included (82). Another study compared combined targeted therapy in the neoadjuvant plus adjuvant setting with physicians’ standard of care in 21 patients with resectable stage III or oligometastatic stage IV melanoma, with only one patient with AM included in the standard of care arm (83). Therefore, no definitive conclusions can be made.

## 7 Future directions in treatment

### 7.1 Immunotherapy

While ICIs have demonstrated significant efficacy in the treatment of advanced CM, their effectiveness is limited in the treatment of AM and MM. One possible reason for lower response rates to immunotherapy in MM and AM may be the mutational burden of the tumor. Typically, the quantity of tumor mutations correlates with the development of neoantigens, leading to an increased response to immunotherapy. AM and MM have a higher incidence of chromosomal structural aberrations and copy number variations and a lower mutational burden than CM, most likely because they are less associated with UV-radiation exposure (5, 20, 22). Resistance to checkpoint inhibitors was linked to NRAS mutations, TP53 mutations, and NF2 deletions, whereas MYC (myelocytomatosis) and RPS6KB1 (ribosomal protein S6 kinase beta-1) amplifications were more prevalent in those who responded to these therapies (112). Challenging these observations, a meta-analysis suggested that NRAS mutations in CM increased the likelihood of partial or complete response to ICI (113).

A potentially effective strategy to overcome immune evasion and prevent resistance to immunotherapy involves combining immunotherapies that aim at various stages of the cancer-immunity cycle. By targeting multiple mechanisms through which malignant cells evade immune surveillance, combination regimens can produce synergistic effects on anti-tumor outcomes while enhancing long-term survival. Several ongoing clinical trials are examining the effectiveness and safety of different combination regimens in patients with acral or mucosal melanoma (114).

Immune cells associated with AM expressed a range of immune checkpoints, such as PD-1, CTLA-4, Lymphocyte-activation gene 3 (LAG-3), V-domain immunoglobin suppressor of T cell activation (VISTA), T cell immunoreceptor with immunoglobulin and ITIM domain (TIGIT), T cell immunoglobulin and mucin domain 3 (TIM-3), and Adenosine A2A receptor (ADORA2), as evidenced by a small single-cell analysis study of primary and metastatic AM (115). In contrast to CM, the expression of VISTA and ADORA2 is higher in AM, while TIGIT expression remains unchanged. TIM-3 (T cell immunoglobulin and mucin domain-containing protein 3) expression is present in 29.2% of myeloid cells, but was significantly lower in AM compared to CM. This indicates less identification of effector CD8 T cells and natural killer cells and a lack of γδ T cells in AM as compared to non-acral melanoma. VISTA, ADORA2, TIGIT, and TIM-3 may thus be considered immune checkpoints with significant potential for future research in the field of AM (115).

LAG-3, also known as CD223, is a CD4-related cell membrane protein that is up-regulated in activated T cells. It binds to MHC class II molecules with high affinity (116). LAG-3 is over-expressed on the membrane of tumor-infiltrating lymphocytes (TILs), and inhibiting LAG-3 leads to a more effective immune-mediated response against tumors (117, 118). LAG-3 and PD-1 are two distinct ICIs that contribute to the depletion of T cells. The RELATIVITY-047 trial evaluated the safety and efficacy of combining relatlimab, a blocking antibody for LAG-3, and nivolumab for treating previously treated melanoma patients. This phase II/III study included 714 patients with previously untreated metastatic or unresectable melanoma, including 41 with AM and 23 with MM, who were treated with either the combination of relatlimab and nivolumab or nivolumab alone. The median PFS was 10.1 months (95% CI, 6.4 to 15.7) with relatlimab-nivolumab in comparison to 4.6 months (95% CI, 3.4 to 5.6) with nivolumab. The safety profile was acceptable. Patients with AM or MM had better outcomes with relatlimab-nivolumab than with nivolumab (46).

TIGIT is an immune checkpoint inhibitor that is upregulated in CD8+ tumor antigen-specific T cells and tumor-infiltrating lymphocytes (TILs) in melanoma patients. It is also co-expressed with PD-1 and binds to CD155 and CD112 ligands. These ligands are present on tumor cells and antigen-presenting cells in the tumor microenvironment. The TIGIT pathway regulates tumor recognition mediated by T cells and natural killer cells both *in vivo* and *in vitro* (119). Therefore, dual blockade of PD-1/TIGIT effectively enhances the expansion and function of CD8+ T cells that are specific for tumor antigens *in vitro*, which leads to tumor rejection in mouse models (119, 120). Clinical trials investigating these combinations in advanced melanoma are ongoing (NCT04305041 and NCT04305054), but patients with MM are excluded. One trial in the adjuvant setting (NCT05665595) is also ongoing but, again, patients with MM are excluded.

TIM-3 upregulation is associated with resistance to anti-PD-1/PD-L1 therapy that is acquired under treatment (121). However, T cells expressing both TIM-3 and PD-1 are more dysfunctional than those expressing PD-1 alone (122). In addition, the MEK inhibitor trametinib can potentially increase TIM-3 expression, leading to a decline in CD8+ T cells. Conversely, an anti-TIM-3 monoclonal antibody can increase CD8+ T cell anti-tumor activity and counteract trametinib-induced depletion of T and NK (Natural Killer) cells in the immune microenvironment. Therefore, combining trametinib and anti-TIM-3 agents may be an option to consider (123). Ongoing clinical trials are investigating the efficacy of a combined blockade of TIM-3 and PD-1 in different types of tumors, particularly in the advanced and neoadjuvant setting (NCT03708328, NCT04370704, and NCT04139902). However, again, patients with MM are excluded.

### 7.2 Tyrosine kinase inhibitors

Tyrosine kinase inhibitors (TKIs), including sorafenib, lenvatinib, imatinib, sunitinib, pazopanib, and axitinib, are being evaluated in patients with AM and MM. Based on the results discussed above (16, 100, 101, 103, 104, 111), these maybe alternative therapeutic options for patients harboring KIT mutations. Combination therapies have shown the most promise (124, 125). Ongoing clinical trials are evaluating the efficacy of several tyrosine kinase inhibitors in AM and MM, including NCT03991975, NCT00788775 and NCT03955354.

### 7.3 Adoptive cell therapy

Tumor-infiltrating lymphocyte-adoptive cell therapy (TILs-ACT) is currently under investigation as a potential treatment alternative for melanoma patients who do not benefit from immunotherapy. This alternative may be particularly attractive in patients who already experienced progressive disease under ICI, namely AM and MM. The process involves isolating TILs from the tumor tissue, expanding them using stimulation with interleukin-2 (IL-2), and reinfusing them into patients after a lymph-depleting chemotherapy in combination with IL-2 therapy (126, 127). This treatment demonstrated an impact on tumor response in Japanese patients with AM or MM who had progressive disease under or after ICI treatment (128).

Recent results from 12 patients included in the phase 2 trial C-144-01 were presented. Here patients with advanced mucosal melanoma who had progressed after anti-PD-1/PD-L1 therapy were treated with lifileucel, a one-time autologous TIL cell therapy. After receiving lymphodepleting chemotherapy, patients received a single infusion of lifileucel, and up to 6 doses of high-dose IL-2. The ORR was 50% (95% CI 21-79%). With a median study follow-up of 35.7 months, the median duration of response was not reached (NR; 95% CI: 12.5 months-NR), the median PFS was NR (95% CI: 1.4 months-NR) and the median OS was 19.4 months (95% CI: 7.9-NR) (129).

The primary limitations of this strategy lie in the technology required for the TILs isolation and expansion, the costs, and time required. Ongoing trials are exploring the use of adoptive T-cell therapy together with checkpoint inhibitors (130).

### 7.4 Engineered lymphocytes for ATC

Chimeric antigen receptors (CARs) are constructed through the fusion of the antigen-binding domain, which is typically derived from the variable regions of antibodies, with signaling domains of the T-cell receptor (TCR) as well as with different co-stimulatory molecules. Anti-GD (Ganglioside D)2/4-1BB CAR T cells are capable of binding to antigens on cancer cells, which activates T cells (131). A preclinical study conducted in China evaluated the ability of these cells to kill ganglioside GD2+ melanoma cells using lesion samples from 288 melanoma patients. Of those samples, 49.3% had positive ganglioside GD2 staining. GD2 expression was more prevalent (50.0% and 56.3%) in acral and mucosal melanomas, respectively, in contrast to chronic sun-induced damage (CSD) (14.3%) and non-CSD (33.3%) melanomas. Patients with ganglioside GD2 expression had a significantly shorter median OS compared to those without ganglioside GD2 expression, with a difference of 31 months versus 47.1 months, respectively (132). Further investigation is needed to evaluate the benefit of this therapy in patients with AM and MM.

### 7.5 Antibody-drug conjugates

Antibody-drug conjugates (ADCs) combine a monoclonal antibody (mAb) with a cytotoxic agent to selectively target tumor cells that overexpress associated tumor-associated antigens (TAAs) (133). *In vitro* analysis demonstrated significantly higher human epidermal growth factor receptor (HER)3 expression in AM cells compared to normal epidermal melanocytes. Additionally, AM cells were sensitive to the cytotoxic component of a HER3-targeted antibody-drug conjugate. These findings indicate that HER3 could serve as a promising novel therapeutic target for AM (134).

### 7.6 Tebentafusp

Tebentafusp is a bispecific protein that employs a high-affinity T cell receptor with an anti-CD3 effector to redirect T cells to target cells that are positive for glycoprotein 100 (Gp-100). Gp-100 is a transmembrane glycoprotein that is highly expressed in melanocytes and melanoma cells. The sensitivity and relative specificity of HMB (Human Melanoma Black)-45, which identifies Gp-100, is an effective indicator of melanoma. In a study of 20 patients with AM, it was reported that 80% of AMs were positively stained with HMB-45 (135). Additionally, the results of a phase I/II trial involving 84 patients with metastatic melanoma (1 with AM and 1 with MM) demonstrate that tebentafusp effectively stimulates anti-tumor immune responses (136).

A Phase I/II clinical trial is underway to evaluate the safety and efficacy of IMC-F106C, an anti-cancer immunomobilizing monoclonal T-cell receptor. The trial is enrolling adult patients with PRAME-positive tumor-associated antigen who have failed standard therapy (relapse or intolerance). In this trial, IMC-F106C is being tested alone or in combination with chemotherapy or tebentafusp or in combination with atezolizumab and pembrolizumab (NCT04262466).

### 7.7 Peptide vaccines and mRNA vaccines

An immunomodulatory vaccine targeting IDO/PD-L1 in combination with nivolumab in metastatic melanoma showed comparable tolerability to nivolumab monotherapy in a phase 1/2 trial. In terms of efficacy, the study demonstrated an ORR of 80% (CI, 62.7 to 90.5%), a median PFS of 26 months (CI, 15.4 to 69 months), and a median OS that was not reached after a median follow-up of 22.9 months (137). The ongoing phase III trial (IOB-013/KN-D18) is evaluating the safety and efficacy of IO102-IO103 dual-antigen with pembrolizumab for first-line treatment in advanced melanoma patients. This two-arm randomized study allocates patients in a 1:1 ratio to receive either dual-antigen IO102-IO103 with pembrolizumab or pembrolizumab alone (NCT05155254).

### 7.8 Neoadjuvant treatment

Neo-adjuvant studies and studies evaluating peri-operative immunotherapy have shown a significant association between pathologic response rates, particularly major pathologic response, and survival outcomes including RFS and distant metastases-free survival (DMFS) (78, 80, 81, 84). However, no information is available regarding the benefit on different subtypes of melanoma.

Further clinical trials are ongoing to evaluate the efficacy of pembrolizumab and vibostolimab (anti-TIGIT; arm 1), pembrolizumab + gebasaxturev (coxsackievirus A21; arm 2), or pembrolizumab alone (arm 3) followed by adjuvant pembrolizumab, in a cohort of 66 patients with stage IIIB-D melanoma as part of phase 1/2 KEYMAKER-U02 substudy 02C (NCT04303169), but MM is an exclusion criteria. The results available so far show in arm 1 showed a pathologic complete response (pCR) rate of 38% (95% CI, 20-59), in arm 2 the pCR rate was 28% (12-49), and in arm 3 it was 40% (16-68). Median RFS and EFS were not reached in any treatment group. ORR was 50% (95% CI, 30-71), 32% (15-54), and 27% (8-55) in arm 1, arm 2, and arm 3, respectively. Treatment with neoadjuvant pembrolizumab in combination with either vibostolimab, gebrolizumab, or on its own, followed by adjuvant pembrolizumab, exhibited a manageable safety profile and promising antitumor activity in patients suffering from stage IIIB-D melanoma. Of all the combination treatments evaluated, the pembrolizumab and vibostolimab combination displayed the most promising results (138). However, there is no information on the inclusion of patients with AM and their outcomes.

Numerous neoadjuvant clinical trials for both AM and MM employing various treatment approaches are currently underway (NCT05545969, NCT03313206, NCT04622566, NCT05512481, NCT04331093).

## 8 Multidisciplinary team

All patients with melanoma, but particularly those with AM and MM should be treated in centers with high volume and experience in managing these tumor entities. Moreover, these cases should be discussed in a multidisciplinary tumor board comprising, among others, dermatologists, surgical oncologists, plastic surgeons, medical oncologists, radiologists, pathologists, radiation oncologists, speech therapists, physiotherapists, rehabilitation specialists, psychologists, social workers, and palliative and supportive care specialists. The purpose of the multidisciplinary team is to provide patients with the latest and individualized treatment, as well as essential assistance in managing the obstacles associated with diagnosis, therapy, and recovery.

## 9 Conclusion

Patients with AM and MM present unique clinical and pathological characteristics that make them less responsive to currently available therapies compared to patients with CM. As a result, there is a pressing need to investigate additional therapeutic options, and this involves the inclusion of AM and MM patients in prospective clinical trials.

Further exploration of treatments targeting other immune checkpoints, including LAG-3, VISTA, TIGIT, TIM-3, ADORA2, as well as therapies involving TIL (tumor-infiltrating lymphocytes), holds promise for delivering survival benefits to this subgroup of patients. The consideration and provision of personalized therapies, guided by NGS (next-generation sequencing) analysis, should be actively offered by the treating physicians, especially when patients do not experience significant benefits from currently available immune checkpoint therapies. Expanding research efforts in these specific subtypes can potentially lead to more effective treatments tailored to their distinct characteristics.

## Author contributions

AF: Writing – original draft, Writing – review & editing. TA: Writing – review & editing.
